# Arterial stiffness and the non-dipping pattern in type 1 diabetes males with and without erectile dysfunction

**DOI:** 10.1038/s41598-023-33315-8

**Published:** 2023-05-04

**Authors:** Michał Kulecki, Dariusz Naskret, Mikolaj Kaminski, Dominika Kasprzak, Pawel Lachowski, Daria Klause, Maria Kozlowska, Justyna Flotynska, Aleksandra Uruska, Dorota Zozulinska-Ziolkiewicz

**Affiliations:** Department of Internal Medicine and Diabetology, Poznan University of Medical Sciences, ul. Mickiewicza 2, 60-834 Poznań, Poland

**Keywords:** Arterial stiffening, Type 1 diabetes

## Abstract

Arterial stiffness (AS) and non-dipping pattern are early predictors of cardiovascular diseases but are not used in clinical practice. We aimed to assess if AS and the non-dipping pattern are more prevalent in the erectile dysfunction (ED) group than in the non-ED group among subjects with type 1 diabetes (T1DM). The study group consisted of adults with T1DM. Aortic pulse wave velocity (PWV Ao)—a marker of increased AS, central systolic blood pressure, and heart rate (HR) were measured with a brachial oscillometric device (Arteriograph 24). Erectile dysfunction (ED) was assessed by the International Index of Erectile Function-5. A comparison between the groups with and without ED was performed. Of 34 investigated men with T1DM, 12 (35.3%) suffered from ED. The group with ED had higher mean 24 h HR (77.7 [73.7–86.5] vs 69.9 [64.0–76.8]/min; p = 0.04, nighttime PWV Ao (8.1 [6.8–8.5] vs 6.8 [6.1–7.5] m/s; p = 0.015) and prevalence of non-dipping SBP Ao pattern (11 [91.7] vs 12 [54.5]%; p = 0.027) than individuals without ED. The presence of ED detected a central non-dipping pattern with a sensitivity of 47.8% and a specificity of 90.9%. The central non-dipping pattern was more prevalent and the nighttime PWV was higher in T1DM subjects with ED than in those without ED.

## Introduction

About 10% of people with diabetes have type 1 diabetes mellitus (T1DM)—the disease caused by autoimmune pancreatic β-cells destruction, therefore it must be treated with exogenous insulin injections^1^. The most common cause of mortality among people with T1DM is cardiovascular disease (CVD).

Arterial stiffness (AS)—progressing rigidity of the arterial walls—is an early predictor of cardiovascular complications, cardiovascular mortality, and all-cause mortality. It allows predicting cardiovascular risk even among people without hypertension and other cardiovascular risk factors^2^. People with T1DM have significantly higher AS than healthy subjects of the same age. Not only the raw value of the blood pressure (BP) is important but also its circadian rhythm^3^. It was proved that non-dippers (people with a lack of or reduction of nocturnal BP fall) have a significantly greater frequency of cardiovascular events than dippers, even if they are normotensives^4^. The dipping pattern is usually marked using peripheral BP. Nevertheless, central compared with brachial BP is more strongly associated with preclinical organ damage^5^. Unfortunately, central hemodynamic measurement is complicated and needs special and expensive devices, inaccessible to most physicians.

Another common problem in adults with T1DM is erectile dysfunction (ED)—“a persistent disorder in achieving and maintaining an adequate erection for satisfactory sexual performance”^6^. In contrary to hemodynamic measurements, ED could be easily investigated by every physician using a simple questionnaire—International Index of Erectile Function-5 (IIEF-5)^7^. Maiorino et al.^8^ revealed that the prevalence of ED is significantly higher (37%) in young men aged 18–35 years with T1DM compared with controls (6%). ED increases the risk of future cardiovascular events and all-cause mortality^9,10^. Some studies revealed an association between increased arterial stiffness, non-dipping pattern, and ED^9,11–14^. However, this issue was never investigated among people with T1DM which differs in many ways from the general population. Previous studies included older males, often with pharmacologically treated hypertension, many other cardiovascular risk factors, and severe or moderate ED. No research investigated the relationship between central BP dipping pattern and ED.

This study aimed to assess if AS and the non-dipping pattern are more prevalent in the erectile dysfunction (ED) group than in the non-ED group among subjects with type 1 diabetes (T1DM).

## Material and methods

### Data collection

This study is based on data from the *Poznań Atherosclerosis in Adult Patients with long-term* Type 1 Diabetes Mellitus Study (PARADISE T1DM Study) which was conducted according to the decision of the Ethical Committee of Poznan University of Medical Sciences (approval No. 67/19). Our study conforms to the principles outlined in the Declaration of Helsinki. Written informed consent was obtained from all participants before inclusion in the study.

The participants of the study were male patients who were under the care of the Department of Internal Medicine and Diabetology. They were consecutively invited to participate in our research from February 2019 to March 2020. The inclusion criteria were: age between 18 and 45 years, T1DM confirmed in the past by positive antibodies with at least a 5-year duration, no less than 70% successful blood pressure measurements, and at least 24 AS available results. We excluded subjects with CVD, hypertension (or using antihypertensives), malignancy, chronic kidney disease (stages 2–5), sleep apnea, history of urological procedures, TSH beyond the normal range, severe or moderate ED ﻿(Supplementary Table 1)﻿.

Initially, every participant had to fill out two forms including basic information on the course of illness, diabetic complications, coexisting diseases, and lifestyle. On admission, every participant underwent anamnesis and a standard physical examination. Specific investigations were performed to detect diabetic complications: urinary albumin excretion, serum creatinine, fundoscopy after mydriasis, tactile sensation using a 10 g monofilament, vibration sensation using a 128 Hz tuning forks, temperature sensation using a rod with two different ends^1^. We also investigated basic anthropometric parameters.

Blood samples were collected from all subjects in the morning, after an 8–12-h overnight fast. The following laboratory results were obtained: lipid profile, thyroid-stimulating hormone, creatinine, transaminases, and C-reactive protein. The low-density lipoprotein cholesterol (LDL-C) level was estimated by the Friedewald formula^15^. HbA1c was evaluated with a turbidimetric inhibition immunoassay (Cobas 6000, Roche Diagnostics).

We assessed the presence of ED by the polish version of IIEF-5 which was used in previous studies^16,17^. The form consists of five questions that allow assessing the presence and severity of ED, from mild through mild-moderate and moderate to severe. Participants were included in two subgroups—with or without ED with a cut-off point of 22 points^7^.

Aortic pulse wave velocity (PWV Ao), aortic augmentation index (AIX Ao), aortic systolic blood pressure (SBP Ao), brachial systolic blood pressure (SBP Br), brachial diastolic blood pressure (DBP Br), and heart rate were measured with a non-invasive brachial oscillometric device—Arteriograph 24 (TensioMed Ltd., Budapest, Hungary). Its operating principle is detecting and processing oscillations on the arm cuff by a special high-fidelity sensor during a complete occlusion of the brachial artery^18^. PWV Ao is calculated automatically by dividing the estimated length of the aorta by half of the return time of the pulse wave. A non-dipping pattern was defined as a nighttime systolic BP (brachial or aortic) fall of less than 10%. Arteriograph 24 was validated using invasive and non-invasive methods of AS assessment^18,19^.

Arteriograph 24 was programmed using TensioWin software to make a measurement every 30 min daily and every 1 h nightly for the next 24 h. Participants were instructed to start manual measurement in case of failed automatic measurement.

Arteriograph performs a series of measurements within 8 s and counts average value and standard deviation. Pulse wave velocity standard deviation within 8 s (PWV SD 8 s) is a quality indicator. We rejected all results with PWV SD 8 s above 1 m/s^20^. We assumed that successful measurement meant at least 70% of available blood pressure results.

Each participant was asked to fill out another questionnaire concerning hour-by-hour activity during 24 h of measurement including physical activity, number of cigarettes, number of (caffeine-containing) coffee cups, body position at the moment of measurement, glycemia, and insulin dosages. The time of sleeping was also marked and used to define daytime and nighttime.

### Data analysis

We used the custom code of the R-programming language (version 3.6.1.; Vienna, R Project) for statistical analysis. The categorical data are presented as numbers (percentage) while numerical as a median (lower quartile to upper quartile). The study group (individuals with ED vs. those without ED) was compared using the Chi-square test and the Mann–Whitney U test. Wet set p-value as two-sided.

We correlated the total score of IIEF-5, as well as the scores (1–5) of all five questions with the outcomes of the Arteriograph measurement. For this purpose, we used a *corrplot* package of R to visualize the R Spearman rank correlation test^21^.

The receiver operating characteristic (ROC) curve was investigated to find the optimal IIEF-5 points value to discriminate individuals with the non-dipping pattern from those with a dipping pattern. The optimal cut-off point was established using Youden's index. We used the *ROCit* package of R^22^.

### Ethical approval

The study was approved by the Ethical Committee of Poznan University of Medical Sciences (nr of consent 67/19).

## Results

### General characteristics

We recruited 46 subjects, but 12 of them had to be excluded because of the insufficient number of successful hemodynamic measurements (9) or incomplete data from the questionnaires (3). We investigated a total of 34 adult men, with age 30.5 (25.0–36.0) and diabetes duration of 13.0 (8.0–21.0) (Table 1). Twelve (35.3%) individuals had an IIEF-5 total score below 22 points and thus were classified as a group with ED. Mild ED had nine (26.5%), three (8.8%) had mild-moderate ED, and no subject had moderate nor severe ED. Entities with ED did not differ significantly in basic characteristics and 24 h-measurement conditions in comparison with those without ED (Table 1). Both groups differed in triglyceride level (ED vs. Non-ED) (1.5 [1.2–1.9] vs 0.9 [0.7–1.3] mmol/l; p = 0.003).

**Table 1:** Comparison of groups with and without self-declared erectile dysfunction (ED) according to The International Index of Erectile Function Questionnaire (IIEF-5). Data presented as median (IQR)/n(%).

Feature	All participants n = 34 (100.0%)	ED group n = 12 (35.3%)	Non-ED group n = 22 (64.7%)	p-value
**General**				
Age [years]	30.5 (25.0–36.0)	32.5 (24.5–39.5)	30.0 (25.0–35.0)	0.63
Diabetes duration [years]	13.0 (8.0–21.0)	15.0 (7.5–23.0)	12.0 (8.0–20.0)	0.71
BMI [kg/m^2^]	26.4 (22.8–28.2)	26.8 (22.8–28.8)	26.4 (22.8–28.0)	0.79
WHR	0.9 (0.9–0.9)	0.9 (0.9–1.0)	0.9 (0.9–0.9)	0.19
**Comorbidities**				
At least one diabetic complication n (%)	18 (50%)	9 (80.0%)	9 (40%)	0.06
Diabetic retinopathy n (%)	8 (23.5%)	5 (41.7%)	3 (13.6%)	0.07
Diabetic nephropathy n (%)	1 (2.9%)	1 (8.3%)	0 (0.0%)	0.17
Diabetic neuropathy n (%)	14 (41.2%)	6 (50.0%)	8 (36.4%)	0.44
**Drugs**				
Continuous Subcutaneous Insulin Infusion n (%)	7 (20.6%)	3 (25.0%)	4 (18.2%)	0.64
Daily insulin intake [insulin units/day/kg]	0.5 (0.4–0.6)	0.6 (0.5–0.7)	0.5 (0.4–0.6)	0.23
Metformin n (%)	5 (14.7%)	2 (16.7%)	3 (13.6%)	0.81
**Lifestyle**				
Current smoker n (%)	11 (32.4%)	3 (25.0%)	8 (36.4%)	0.50
Packyears	0.1 (0.0–6.0)	1.3 (0.0–9.2)	0.0 (0.0–6.0)	0.58
Alcohol intake [units/week]	1.0 (0.5–4.0)	2.0 (0.0–4.5)	1.0 (1.0–3.0)	0.99
Shift work n (%)	13 (38.2%)	5 (41.7%)	8 (36.4%)	0.76
Sleeping [hours/day]	7.0 (6.0–8.0)	7.0 (6.0–8.0)	7.0 (6.0–7.5)	0.79
Physical work [hours/day]	3.0 (0.8–8.0)	6.0 (1.0–8.5)	3.0 (0.0–8.0)	0.34
Sport activity [hours/week]	2.9 (0.0–8.0)	2.0 (0.0–7.5)	4.3 (0.0–8.0)	0.66
Average_sitting_per_week	5.5 (3.9–7.4)	5.2 (4.0–5.8)	6.1 (3.9–7.6)	0.51
**Lab findings** **and others**				
HbA1c [%]	9.0 (7.7- 10.1)	9.3 (8.5–10.1)	8.4 (7.0–10.1)	0.26
Total cholesterol [mmol/l]	4.7 (4.1–5.1)	5.0 (4.4–5.8)	4.3 (4.0–4.9)	0.058
LDL-C [mmol/l]	2.4 (2.0–2.8)	2.7 (2.3–3.3)	2.4 (1.7–2.8)	0.10
HDL-C [mmol/l]	1.6 (1.4–1.8)	1.4 (1.3–1.7)	1.7 (1.5–1.8)	0.18
Triglycerides [mmol/l]	**1.2 (0.8–1.5)**	**1.5 (1.2–1.9)**	**0.9 (0.7–1.3)**	**0.003**
ALT [UI/l]	17.0 (13.0–21.0)	19.5 (13.5–25.5)	16.0 (13.0–20.0)	0.23
AST [UI/l]	17.0 (15.0–19.0)	17.5 (16.0–21.0)	16.0 (15.0–18.0)	0.11
ACR [mg/mmol]	4.5 (3.0–10.0)	8.5 (4.0–16.5)	3.0 (3.0–6.0)	0.053
C-reactive protein [nmol/l]	10.0 (6.2–16.7)	9.5 (6.5–13.5)	10.1 (5.2–19.0)	0.82
Estimated glucose disposal rate [mg/kg/min]	8.4 (7.4–8.9)	8.0 (6.8–8.5)	8.6 (7.5–9.2)	0.09
**Activity during day of measurement**				
Time of physical activity [min]	15.0 (0.0–40.0)	10.0 (0.0–30.0)	25.0 (0.0–60.0)	0.33
Number of cigarettes [n]	0.0 (0.0–6.0)	0.0 (0.0–3.5)	0.0 (0.0–6.0)	0.90
Number of coffee cups [n]	2.0 (1.0–3.0)	2.0 (1.0–3.0)	2.0 (0.0–3.0)	0.99
Mean glycemia [mmol/l]	7.8 (6.9–9.4)	8.6 (7.2–10.3)	7.7 (6.8–8.8)	0.15
SD of glycemia [mmol/l]	2.8 (2.4–3.5)	3.0 (2.6–3.3)	2.7 (1.9–3.5)	0.23
Total insulin dose [units]	42.5 (36.5–50.0)	46.5 (37.9–56.0)	40.8 (36.5–48.0)	0.34
Number of measurements in sitting position [n]	9.5 (7.0–14.0)	13.5 (7.0–17.0)	9.0 (7.0–13.0)	0.28
Number of measurements in standing position [n]	5.0 (4.0–7.0)	4.0 (4.0–5.5)	6.0 (4.0–8.0)	0.29
Number of measurements in lying position [n]	12.0 (10.0–16.0)	13.0 (11.5–20.5)	11.5 (9.0–14.0)	0.11

Significant values are in [bold].

*ACR* albumin to creatinine ratio, *ALT* alanine transaminase, *AST* aspartate transaminase, *BMI* body mass index, *HbA1c* glycated hemoglobin, *HDL-C* high-density lipoprotein, *LDL-C* low-density lipoprotein, *WHR* waist-to-hip ratio.

### Hemodynamic parameters

The 24 h-measurement revealed the difference between the study groups in mean 24 h heart rate (77.7 [73.7–86.5] vs 69.9 [64.0–76.8]/min; p = 0.037), nighttime heart rate (71.0 [57.0–77.3)] vs 57.0 [51.2–63.8]/min; p = 0.044), nighttime PWV Ao (8.1 [6.8–8.5] vs 6.8 [6.1–7.5] m/s; p = 0.015) and standard deviation of nighttime AIX Ao (7.5 [5.1–9.9] vs 5.8 [4.5–6.5]%; p = 0.037). All measured parameters did not differ significantly during the day. 24-h PWV Ao tended to be increased in the ED group compared to the non-ED group (8.6 [7.7–8.9] vs 7.7 [6.8–8.2]; p = 0.058). The total score of IIEF-5 was negatively associated with nighttime PWV Ao (Rs = − 0.44; p < 0.01), 24 h heart rate (Rs = − 0.47; p < 0.01), nighttime heart rate (Rs = − 0.42; p = 0.01) and the standard deviation of nighttime PWV Ao (Rs = − 0.39; p = 0.02). The average PWV SD 8 s for the whole group was below 0.7 m/s (0.4 during the day and 0.3 during the night). It indicates a high quality of AS measurements (Table 2).

**Table 2:** Comparison of groups with and without self-declared erectile dysfunction (ED) according to The International Index of Erectile Function Questionnaire (IIEF-5). Data presented as median (IQR)/n(%).

Feature	All participants n = 34 (100.0%)	ED group n = 12 (35.3%)	Non-ED group n = 22 (64.7%)	p-value
**24 h-measurements**				
SBP Ao [mmHg]	119.2 (116.8–127.9)	119.7 (115.9–129.2)	119.2 (116.8–126.6)	0.99
SBP Ao SD [mmHg]	13.1 (11.0–16.0)	13.3 (10.9–17.8)	12.9 (11.3–16.0)	0.99
SBP Br [mmHg]	132.8 (128.2–140.2)	132.8 (126.6–142.5)	132.8 (128.5–140.2)	0.96
SBP Br SD [mmHg]	13.0 (10.2–15.0)	12.7 (10.0–20.1)	13.0 (10.2–15.0)	0.93
DBP Br [mmHg]	77.7 (73.8–81.4)	76.7 (72.9–83.9)	78.0 (74.9–80.4)	0.93
DBP Br SD [mmHg]	11.2 (9.3–13.9)	11.1 (9.0–14.5)	11.2 (9.3–13.9)	0.96
Pulse pressure [mmHg]	55.9 (50.7–63.7)	56.2 (49.9–64.4)	55.9 (50.7–62.5)	0.99
Heart rate [/min]	**72.6 (65.6–79.7)**	**77.7 (73.7–86.5)**	**69.9 (64.0–76.8)**	**0.037**
Heart rate SD [/min]	10.0 (8.7–14.3)	10.0 (8.8–14.5)	9.9 (8.2–14.3)	0.87
AIX Ao [%]	14.2 (10.4–17.9)	14.3 (11.6–17.3)	13.8 (9.3–17.9)	0.66
AIX Ao SD [%]	7.1 (5.8–9.1)	7.8 (6.9–8.8)	6.4 (5.7–10.0)	0.25
AIX Br [%]	− 46.6 (− 53.8 to − 39.0)	− 46.0 (− 51.4 to − 40.1)	− 47.6 (− 55.9 to − 39.0)	0.58
AIX Br SD [%]	14.5 (11.7–17.9)	15.4 (13.6–17.5)	12.8 (11.5–19.8)	0.34
PWV Ao [m/s]	7.9 (7.2–8.7)	8.6 (7.7–8.9)	7.7 (6.8–8.2)	0.058
PWV Ao SD [m/s]	1.1 (0.8–1.2)	1.2 (0.8–1.3)	1.0 (0.9–1.1)	0.61
PWV SD 8 s [m/s]	0.4 (0.3–0.5)	0.5 (0.3–0.5)	0.4 (0.3–0.4)	0.29
**Daytime measurements**				
SBP Ao [mmHg]	123.9 (118.9–130.3)	121.3 (115.6–132.7)	124.8 (119.5–129.2)	0.63
SBP Ao SD [mmHg]	11.8 (10.0–13.7)	11.8 (9.6–13.7)	11.6 (10.0–14.9)	0.85
SBP Br [mmHg]	135.2 (131.7–143.9)	135.2 (127.3–146.9)	135.2 (133.0–143.9)	0.79
SBP Br SD [mmHg]	11.5 (9.3–14.5)	13.0 (9.6–21.0)	10.7 (9.3–14.5)	0.47
DBP Br [mmHg]	79.3 (75.0–84.5)	77.8 (74.2–87.3)	79.7 (76.7–83.4)	0.76
DBP Br SD [mmHg]	10.3 (8.3–12.1)	10.3 (7.9–15.3)	10.3 (8.3–11.9)	0.85
Pulse pressure [mmHg]	55.7 (51.2–65.0)	56.7 (50.5–65.2)	55.7 (51.2–64.8)	0.96
Heart rate [/min]	76.4 (68.4–84.3)	81.2 (75.7–88.6)	73.5 (67.9–81.8)	0.094
Heart rate SD [/min]	9.7 (7.5–12.9)	11.2 (8.0–14.4)	8.7 (7.5–12.6)	0.19
AIX Ao [%]	12.3 (8.6–13.8)	12.1 (9.4–13.9)	12.6 (8.4–13.8)	0.99
AIX Ao SD [%]	6.5 (5.3–7.7)	6.6 (5.4–7.4)	6.5 (5.1–7.8)	0.87
AIX Br [%]	− 50.8 (− 57.4 to − 47.1)	− 50.5 (− 55.7 to − 47.0)	− 51.0 (− 57.7 to − 47.1)	0.90
AIX Br SD [%]	13.3 (10.8–17.0)	13.1 (10.7–14.6)	13.3 (10.8–17.0)	0.56
PWV Ao [m/s]	8.2 (7.3–8.9)	8.9 (7.8–9.3)	8.0 (7.3–8.6)	0.094
PWV Ao SD [m/s]	1.1 (0.9–1.3)	1.2 (0.8–1.4)	1.0 (0.9–1.1)	0.34
PWV SD 8 s [m/s]	0.4 (0.4–0.6)	0.5 (0.4–0.6)	0.4 (0.4–0.5)	0.20
**Nighttime measurements**				
SBP Ao [mmHg]	114.4 (109.6–121.2)	117.0 (110.9–125.7)	113.5 (102.4–118.4)	0.14
SBP Ao SD [mmHg]	10.6 (9.1–12.4)	9.9 (9.0–11.3)	11.3 (9.1–12.9)	0.47
SBP Br [mmHg]	120.8 (116.0–130.9)	129.7 (118.8–133.2)	120.2 (113.4–129.1)	0.11
SBP Br SD [mmHg]	11.5 (7.7–12.5)	9.9 (6.7–12.2)	11.6 (8.2–12.9)	0.42
DBP Br [mmHg]	69.8 (64.9–74.8)	71.4 (69.1–76.3)	68.5 (58.9–72.0)	0.058
DBP Br SD [mmHg]	8.1 (7.1–10.5)	8.0 (6.9–9.4)	8.6 (7.4–10.5)	0.44
Pulse pressure [mmHg]	53.4 (48.3–60.8)	56.8 (48.7–62.2)	52.6 (48.3–57.8)	0.68
Heart rate [/min]	**58.5 (52.4–72.1)**	**71.0 (57.0–77.3)**	**57.0 (51.2–63.8)**	**0.044**
Heart rate SD [/min]	5.2 (4.0–6.8)	5.6 (4.0–9.3)	4.9 (3.9–6.1)	0.26
AIX Ao [%]	17.5 (12.3–22.7)	18.0 (15.7–24.6)	17.4 (11.6–22.0)	0.28
AIX Ao SD [%]	**6.2 (4.7–7.7)**	**7.5 (5.1–9.9)**	**5.8 (4.5–6.5)**	**0.037**
AIX Br [%]	− 39.7 (− 50.0 to − 29.4)	− 38.7 (− 43.4 to − 25.8)	− 39.9 (− 51.5 to − 30.8)	0.26
AIX Br SD [%]	12.3 (9.3–15.8)	14.8 (10.1–19.5)	11.5 (8.9–13.1)	0.048
PWV Ao [m/s]	**7.0 (6.5–8.0)**	**8.1 (6.8–8.5)**	**6.8 (6.1–7.5)**	**0.015**
PWV Ao SD [m/s]	0.6 (0.4–0.8)	0.5 (0.3–0.8)	0.7 (0.4–0.9)	0.42
PWV SD 8 s [m/s]	**0.3 (0.3–0.4)**	**0.4 (0.3–0.6)**	**0.3 (0.3–0.4)**	**0.04**
**Dipping pattern**				
SBP Ao non-dipper n (%)	**23 (67.6%)**	**11 (91.7%)**	**12 (54.5%)**	**0.027**
SBP Br non-dipper n (%)	18 (52.9%)	9 (75.0%)	9 (40.9%)	0.057
Nocturnal SBP Ao fall (%)	**7.2 (**− **0.2 to 13.3)**	**3.6 (− 2.3 to 7.8)**	**9.0 (1.5–18.4)**	**0.040**
Nacturnal SBP Br fall (%)	**9.6 (4.1–13.9)**	**4.3 (3.5–10.2)**	**11.2 (7.3–18.1)**	**0.044**

Significant values are in [bold].

*PWV Ao* aortic pulse wave velocity, *AIX Ao* aortic augmentation index, *AIX Br* brachial augmentation index, *SBP Ao* aortic systolic blood pressure, *SBP Br* brachial systolic blood pressure, *DBP Br* brachial diastolic blood pressure, *SD* standard deviation, *PWV SD 8 s* 8 s standard deviation of pulse wave velocity-indicator of measurement quality.

### Non-dipping pattern

**Figure 1:**
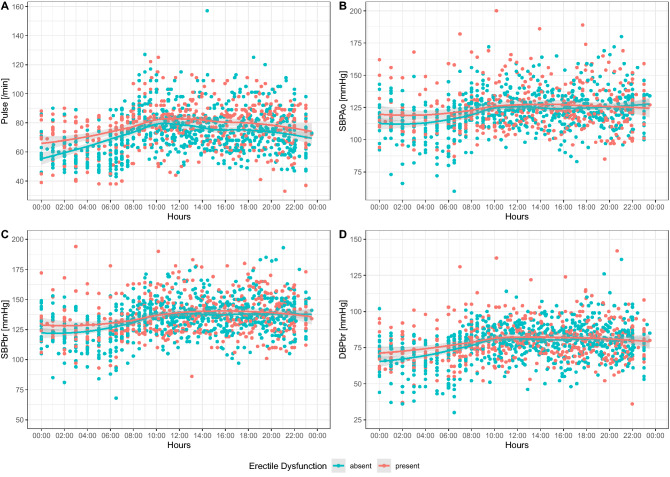
Daily variability of hemodynamic parameters. X-axis: time (h). Y-axis: measured hemodynamic parameter. *SBP Ao* aortic systolic blood pressure, *SBP Br* brachial systolic blood pressure, *DBP Br* brachial diastolic blood pressure.

**Figure 2:**
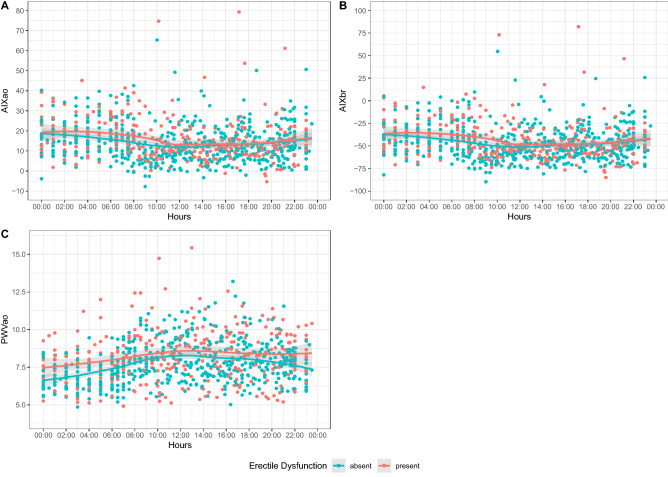
Daily variability of hemodynamic parameters. X-axis: time (h). Y-axis: measured hemodynamic parameter. *PWV Ao* aortic pulse wave velocity, *AIX Ao* aortic augmentation index, *AIX Br* brachial augmentation index.

The presence of a non-dipping pattern based on SBP Ao was significantly more prevalent in the ED group than in the non-ED (11 [91.7%] vs. 12 [54.5%]; p = 0.027). Both SBP Ao (3.6 [-2.3–7.8] vs 9.0 [1.5–18.4] %; p = 0.04) and SBP Br (4.3 [3.5–10.2] vs 11.2 [7.3–18.1] %; p = 0.044) nocturnal falls were significantly lower in ED group than in non-ED group (Table 2) Figs. 1 and 2 show all of the 24-h measurement results in ED group and non-ED group. PWV Ao and heart rate were higher during 24 h of measurement, but the difference became significant only during the night. Diurnal SBP Ao and SBP Br did not differ significantly between the groups.

ROC curve analysis revealed that the IIEF-5 total score of 21 (based on Younden's index) was the best cut-off to diagnose a non-dipping pattern. IIEF-5 total score equal to or lower than 21 (the same cut-off point is used to diagnose ED) suggests the presence of a non-dipping pattern with a sensitivity of 47.8% and a specificity of 90.9%. The area under the curve (AUC) was 0,68 (95% CI 0.50–0.87).

## Discussion

### Main findings

In this study, we analyzed hemodynamic parameters in the two groups of T1DM males—with and without ED. We found that people with T1DM and mild or mild-moderate ED had a significantly higher 24-h heart rate, nighttime PWV Ao, and greater prevalence of central BP non-dipping pattern than those without ED. IIEF-5 form allowed recognition of non-dipping pattern with high specificity but low sensitivity.

To our knowledge, no studies have investigated the relationship between 24-h hemodynamic and ED in the T1DM population yet. A recent study revealed that men with severe but not with moderate or mild ED have an increased risk of CVD and all-cause mortality^23^. Turek et al. observed that among T1DM subjects with a long disease duration (more than 50 years) ED can be a predictor of CVD^24^. In contrary, our study revealed that even young T1DM individuals with shorter disease duration and mild or mild-moderate ED presented a couple of real subtle changes suggesting impaired cardiovascular condition.

### Pulse wave velocity

Association between ED and PWV was observed earlier but never in the T1DM population. Kumagai et al. proved the negative relationship between IIEF5 score and carotid-femoral PWV among 317 Japanese men with comorbidities^11^. Another research revealed that PWV was associated negatively with erection hardness score^25^. Our study showed a tendency to higher 24-h PWV but the difference was significant only for nighttime PWV. The possible explanation is the presence of additional factors that could influence PWV value during the day like physical activity, insulin boluses, and different positions during measurement. This explanation is supported by the average results of PWV Ao standard deviation which was 1.1 m/s for daytime and only 0.6 m/s for nighttime measurements.

### Non-dipping pattern

Among people with diagnosed hypertension, the non-dipping brachial BP pattern was proved as an independent determinant for ED^13^. Another study performed in Sri Lanka among 29 hypertensives with type 2 diabetes mellitus revealed a high (72.4%) prevalence of non-dipping brachial BP pattern^26^. Gourgari et al. revealed that 48% of 25 T1DM subjects presented a non-dipping pattern^27^. In the group of non-dipper hypertensives, people with severe ED had lower dipping BP levels in comparison with those without ED^12^. In our study, more than 90% of ED subjects presented a non-dipping central BP pattern. Kumagai et al. described the relationship between central BP and moderate-to-severe ED. However, we revealed for the first time that the non-dipping central BP pattern is more prevalent in males with mild and mild-moderate ED than in males without ED among individuals with T1DM. We assume both non-dipping pattern and ED may result from autonomic neuropathy which disturbs circadian BP rhythm and autonomic regulation of sexual functions. The diagnosis of a non-dipping pattern has clinical implications. The MAPEC study revealed that the administration of at least one of the antihypertensive drugs at bedtime (versus taking all of the medicines in the morning) reduced the prevalence of non-dipping pattern and significantly decreased cardiovascular morbidity and mortality^28^.

### Heart rate

We found a negative association between the average 24-h heart rate and the total score of IIEF-5. The mean 24 h heart rate was higher in the group with ED than in the non-ED group. It is similar to the results of Kratz et al.^29^. They observed that elevated heart rate—above the median of mean heart rate—was associated with the presence of ED. Increased heart rate is also a cardiovascular risk factor^30^. In cross-sectional study people with T1DM were compared with their monozygotic twins without T1DM and they had significantly higher resting heart rate^31^.

### Pathophysiology

Our research included young men with T1DM. They presented no classic risk factors for ED and CVD but because of T1DM, they had other possible mechanisms to develop both of them. Chronic hyperglycemia results in oxidative stress, endothelial impairment, lack of nitric oxide, and predominance of vasoconstrictive pathways. Tibiriçá et al. proved microvascular impairment in T1DM which follows from a lack of capillary reserve^32^. High glucose levels predispose to the accumulation of advanced glycation end products, preterm atherosclerosis, and thickening of vascular walls which results in arterial stiffness. These changes cause insufficient blood flow in corpora cavernosa^33^. There is a conception that atherosclerosis develops earlier in the penile artery than in coronary arteries because the penile artery is smaller in diameter^9,10^. Hyperglycemia also damages nerves. Somatic neuropathy affects sensory signals from the penis. Autonomic neuropathy results in impaired relaxation of smooth muscles in corpora cavernosa and also weakens the vasodilatation of penile arterioles^34^.

Dyslipidemia is a risk factor for both CVD and ED^1,6^. We found the difference in triglycerides between ED and non-ED group with a higher average value for ED. It is similar to the results obtained by Corona et al.^35^. They proved that elevated triglycerides are associated with arteriogenic ED and future cardiovascular events. The triglyceride level is an important cardiovascular risk factor positively associated with PWV independently of blood glucose level, SBP, and age^36^. This common risk factor may partly explain the difference in PWV Ao between ED and non-ED group.

ED and CVD are both common problems among T1DM individuals. However, we dispose of multiple effective methods to prevent and cure them. The basis should be a healthy lifestyle and intensive glycemic control. Intensive insulin therapy may reduce resting heart rate, risk of ED, a progression of micro-, and macrovascular complications^37–40^. More than 60% of males including those with diabetes mellitus, positively respond to the phosphodiesterase type 5 inhibitors^41^.

### Strengths and practical implications

This is the first study investigating the relationship between 24-h variability of central hemodynamic and ED among T1DM subjects. Our results lead to clinical implications for T1DM individuals. If the presence of ED relates to increased cardiovascular risk indicators, those subjects should be watchfully observed. Physicians in Poland do not have complete access to devices measuring central hemodynamic, but the IIEF-5 questionnaire is available. Unfortunately, only less than 50% of subjects with ED aged 18–40 years talk to doctors about their sexual dysfunction^42^.

### Limitations

Our study has several limitations. Firstly, a small number of participants could not be representative of the T1DM population. Nevertheless, we performed restricted inclusion and exclusion criteria. Moreover, we analyzed not 34 single measurement results but more than nine hundred for the whole group. Secondly, ED diagnosis was based on the IIEF-5 questionnaire and did not include ultrasound doppler and testosterone levels. Thirdly, none of the participants had PWV above 10 m/s, therefore it was impossible to assess the prevalence of AS^10^. Lastly, it was a cross-sectional study, and we cannot state any cause-and-effect relationship.

## Conclusion

Among men with T1DM, males with ED have increased nighttime PWV Ao, elevated heart rate, and higher prevalence of the non-dipping central BP pattern than males without ED. Even mild and mild-moderate ED is related to significant changes in central hemodynamics. ED assessment can be an easy tool for identifying T1DM male subjects with non-dipping pattern and increased cardiovascular risk. Further studies with a more generous experimental group are needed to confirm those results. 

## Supplementary Information

Supplementary Table 1.

## Data Availability

The data that support the findings of this study are available from the corresponding author upon reasonable request.
