# Supplementary material for: Arterial stiffness and the non-dipping pattern in type 1 diabetes males with and without erectile dysfunction
Source: Sci Rep. 2023 May 4;13:7265. doi: 10.1038/s41598-023-33315-8 (PMC10160017; doi:10.1038/s41598-023-33315-8)
Supplement: Supplementary file 1 — Supplementary Table 1. [file 41598_2023_33315_MOESM1_ESM.pdf]

Title: Arterial stiffness and the non-dipping pattern in type 1 diabetes males with and without erectile dysfunction

Authors:

Michal Kulecki*

Dariusz Naskret*

Mikolaj Kaminski

Dominika Kasprzak

Pawel Lachowski

Daria Klause,

Maria Kozlowska

Justyna Flotynska

Aleksandra Uruska#

Dorota Zozulinska-Ziolkiewicz#

# - equal senior author

* - equal first author

Supplementary table 1.
Inclusion and exclusion criteria of the study

Inclusion criteria	Exclusion criteria
• Conscious written consent<br>• Age between 18-45 years<br>• T1DM confirmed in the past by positive antibodies<br>• At least 5-year duration of T1DM<br>• Complete data<br>• At least 70% of successful blood pressure measurements<br>• At least 24 successful arterial stiffness measurements	• Cardiovascular disease<br>• Heart failure<br>• Diagnosed malignancy<br>• Clinically significant psychiatric disorder<br>• Chronic kidney disease (stages 2-5)<br>• hypo or hyperthyroidism<br>• Alcoholism<br>• Sleep apnoea<br>• History of urological procedures<br>• Hypo- or hyperthyroidism (TSH beyond normal range)<br>• Using of the following medications during last 3 months: antihypertensives, ASA, other antiplatelet drugs, statins, diuretics, phosphodiesterase 5 inhibitors.<br>• Contagious diseases<br>• Severe or moderate ED

T1DM – type 1 diabetes mellitus; TSH - thyroid stimulating hormone; ASA - acetylsalicylic acid; ED – erectile dysfunction.
